# Establishing a screening strategy for non-discriminatory reactive blood donors by constructing a predictive model of hepatitis B virus infection status from a single blood center in China

**DOI:** 10.3389/fpubh.2024.1366431

**Published:** 2024-03-27

**Authors:** Danxiao Wu, Yiqin Hu, Min Wang, Yaling Wu, Jie Dong, Jinhui Liu, Wei Hu

**Affiliations:** ^1^Department of Laboratory, Blood Center of Zhejiang Province, Hangzhou, China; ^2^Key Laboratory of Blood Safety Research of Zhejiang Province, Hangzhou, China

**Keywords:** non-discriminatory reactive, occult hepatitis B infection, nucleic acid testing, predictive model, screening strategy

## Abstract

**Background:**

When employing the transcription-mediated amplification method for screening blood donors, there are some non-discriminatory reactive results which are screening assay reactive but HBV-DNA discriminatory assay negative. This raises concerns regarding the possibility of false positives among donors, which may lead to permanent deferral of blood donors and affect blood supply. This study aimed to elucidate the infection status of these non-discriminatory reactive blood donors and develop and validate a model to predict individualized hepatitis B status to establish an optimal screening strategy.

**Methods:**

Supplementary tests were conducted on initial non-discriminating reactive donations to determine their HBV infection status, including repeat testing, viral load, serological marker detection, and follow-up. Primary clinical variables of the donors were recorded. Based on the Akaike information criterion, a stepwise forward algorithm was used to identify the predictive factors for information and construct a predictive model. The optimal screening strategy was determined through cost-effectiveness analysis.

**Results:**

At the Blood Center of Zhejiang Province, 435 cases of initial non-discriminatory reactive donations were collected over two successive periods and sub-categorized through repeated testing into the following three groups: non-repeated positive group, non-discriminated positive group, and non-repeated HBV-DNA positive group. The HBV discriminatory rate increased after repeated testing (110/435, 25.29%). According to supplementary tests, the HBV-DNA positivity rate was 65.52% (285/435), and occult HBV infection was a significantly different among groups (χ^2^ = 93.22, *p* < 0.01). The HBV serological markers and viral load in the non-repeated positive group differed from those in the other two groups, with a lower viral load and a higher proportion of false positives. The predictive model constructed using a stepwise forward algorithm exhibited high discrimination, good fit, high calibration, and effectiveness. A cost-effectiveness analysis indicated that utilizing repeated discriminatory testing and the predictive model is an extremely beneficial screening approach for non-discriminatory reactive blood donors.

**Conclusion:**

Nearly two-third (65.52%) of the non-discriminatory reactive blood donors were HBV-DNA positive. Our innovative approach of constructing a predictive model as a supplementary screening strategy, combined with repeated discriminatory experiments, can effectively identify the infection status of non-discriminatory reactive blood donors, thereby increasing the safety of blood transfusions.

## Introduction

China is a higher intermediate prevalence area of Hepatitis B infection, with an incidence rate ranging from 5 to 7.99% (1). Therefore, blood screening for HBV is essential in reducing the transmission of infectious diseases by transfusion and is a high priority for blood banks. In addition to the screening strategy based on hepatitis B virus surface antigen (HBsAg) detection, since 2010, nucleic acid testing (NAT) technology has been implemented to simultaneously detect HBV-DNA, HCV-RNA, and HIV-RNA in a few pilot blood banks nationwide, including the Blood Center of Zhejiang Province starting from August 1, 2010 (2).

Currently, blood banks in China mainly use enzyme-linked immunosorbent assay (ELISA) to detect HBsAg, while the main methods for screening hepatitis B virus nucleic acids include polymerase chain reaction (PCR) and transcription-mediated amplification (TMA), which significantly reduce the assay window for immunological assays and exhibit a sensitivity several orders of magnitude higher than that of antigen and/or antibody immunoassays (3). Moreover, the PCR and TMA detection processes are different. The PCR conducts screening tests in a mini-pool NAT (MP-NAT) mode, and if HBV-DNA is positive in MP-NAT, individual NAT (ID-NAT) is performed to determine the reactive status of blood donation, while the TMA uses ID-NAT for mixed project screening tests. If the initial screening is positive, HBV-DNA discriminatory testing is required; however, initially positive donations are considered reactive samples regardless of the discriminatory test results (4).

The TMA method analysis revealed ELISA-negative and non-discriminating reactive blood donors who tested positive in the screening tests but negative in the discriminatory NAT assay. Some studies have reported that these non-discriminating reactive donors may indicate possible occult hepatitis B infection (OBI) with low or fluctuating levels of HBV-DNA in the blood (5, 6), which is defined as the presence of replication-competent HBV-DNA [episomal HBV covalently closed circular DNA (cccDNA)] in the liver and/or HBV-DNA in the blood of people who test negative for HBsAg by currently available assays (7). In our previous follow-up study of 138 repeat blood donors, only 42.03% of the donors exhibited NAT repeat reactivity, with a proportion of 40.00% (52/130) for non-discriminating reactive donors. Moreover, a risk of false negatives in NAT screening for blood donors with low-level HBV viral loads was observed, causing fluctuating HBV viral loads and leading to non-repeat reactive NAT results in the follow-up study (8). This raises the question of whether these non-discriminating reactive blood donors are false positives. According to our previous studies, in the Blood Center of Zhejiang Province, the prevalence of all NAT^+^ELISA^−^ using the TMA method was 2625.06 per million, with a non-discriminating reactive prevalence of 1519.36 per million, accounting for 57.88% (4). Against the backdrop of a general blood shortage, the issue of discarding blood donation and permanent deferral of donors due to false positive blood test results has received double attention (9–11). Therefore, identifying true-positive cases of this type is crucial for maintaining a stable blood supply.

This study conducted supplementary tests on initial non-discriminating reactive blood donations, including repeat testing, viral load testing and serological marker detection. Follow-up was conducted on all blood donors with negative HBV-DNA results in the supplementary experiments to determine their HBV infection status. Additionally, a prediction model was developed and validated to predict the individualized status of hepatitis B infection in these non-discriminatory reactive blood donors to establish their screening strategy.

## Materials and methods

### Blood samples

Blood samples were collected from voluntary blood donors at the Blood Center of Zhejiang Province from July 2012 to June 2014, and from January 2017 to December 2018. Specimens were subjected to NAT using TMA method. All blood donors complied with the “Health Examination Requirements for Blood Donors” and signed an informed consent form authorizing the use of their donated blood for pertinent medical research. All specimens were tested for HBsAg using ELISA with diagnostic reagent kits for hepatitis B surface antigen (HBsAg) from InTec Products (Xiamen, China) and Bio-Rad Laboratories (Shanghai, China). Simultaneously, TMA nucleic acid testing was performed. From July 2012 to June 2014, Procleix^®^ Ultrio^®^ Assay (Grifols Diagnostic Solutions Inc., Emeryville, CA, United States) was used with a lower detection limit for HBV-DNA of 10.4 IU/mL. From January 2017 to December 2018, Procleix^®^ Ultrio Elite^®^ Assay (Grifols Diagnostic Solutions Inc., Emeryville, CA, United States) was used with a lower detection limit for HBV-DNA of 4.3 IU/mL.

### HBsAg^−^NAT^+^ initial non-discriminated sample

A total of 540 initial non-discriminatory reactive blood donations were collected, which were HBsAg-ELISA negative, TMA screening assay reactive but HBV-DNA discriminatory assay negative. Of these, 355 samples were collected from July 2012 to June 2014, and 185 samples were collected from January 2017 to December 2018. Variables including age, gender, native place, vaccination status at birth, ABO and RhD blood type, body mass index (BMI), marital status, education level, occupation, blood donation type, interval time of donation (days), times of donation and types of donors were recorded.

### Nucleic acid repeat experiments

All initial non-discriminatory reactive samples underwent two identical screening and discriminatory tests. Based on the results of the repeat screening and discriminatory tests, they were categorized into three groups (8): (i) non-repeated positive group (NRP), with a repeat screening NAT assay negative and discriminatory NAT assay for non-reactive HBV-DNA; (ii) non-discriminated positive group (NDP), with a repeat screening NAT assay reactive but discriminatory NAT assay non-reactive for HBV-DNA; and (iii) non-repeated HBV-DNA positive group (NR-HBV), with a repeat screening NAT assay reactive and discriminatory NAT assay for HBV-DNA was once reactive but not repeatable. All initially identified non-discriminatory samples were re-tested using the Cobas^®^ TaqScreen MPX Test version 2.0 (Roche Molecular Systems, Inc., South Branchburg, NJ, United States) by Cobas TaqMan analyzer (Roche Diagnostics Company, Shanghai, China) with a minimum detection limit of 2.3 IU/mL for HBV-DNA. Among them, 92 samples were from July 2012 to June 2014, and 5 samples were from January 2017 to December 2018, which did not undergo further serological testing due to insufficient sample volume.

### Supplementary serological experiments

All initial non-discriminated reactive samples were tested for HBV serum markers including HBsAg, hepatitis B surface antibody (anti-HBs), hepatitis B e antigen (HBeAg), hepatitis B e antibody (anti-HBe), and hepatitis B core antibody (anti-HBc). The experiments were performed by electrochemiluminescence immunoassay (ECLIA) with a Cobas e601 analyzer (Roche Diagnostics Company, Shanghai, China) or chemiluminescent immunoassay (CLIA) with an ARCHITECTTM i2000SR analyzer (Abbott Diagnostics, Abbott Park, IL, United States), According to the instructions of the reagents, the negative criteria were HBsAg <0.05 IU/mL, anti-HBs < 10 mIU/mL, HBeAg S/Co value <1.0, anti-HBe S/Co value >1.0, and anti-HBc S/Co value <1.0.

### Fluorescent quantitative PCR experiments

All initial non-discriminated reactive samples were tested for viral load using the fluorescent quantitative PCR method by COBAS^®^ AmpliPrep/COBAS^®^ TaqMan^®^ HBV Test (Roche Molecular Systems, Inc., South Branchburg, NJ, United States). According to the instructions of the reagents, the results were categorized into three types: (i) negative, no HBV-DNA detected; (ii) <12 IU/mL, HBV-DNA detected but below the detection range; and (iii) >12 IU/mL, HBV-DNA detected and above the detection range, with specific values representing the viral load.

### Follow-up of blood donors

Follow-up was conducted for blood donors whose all supplementary test results were HBV-DNA negative at intervals of more than 2 weeks until loss to follow-up, and eight of them were lost to follow-up. Follow-up samples were analyzed for HBV serological markers and HBV-DNA. HBV infection was confirmed when HBV-DNA was detected, or seroconversion was observed during follow-up.

### Identification of HBV infection status in blood donors

Nucleic acid repeat experiments, supplementary serological experiments, viral load assays and follow-up studies were conducted to identify and ascertain the HBV infection status of the donors. We defined blood donors who were HBV-DNA positive but HBsAg negative and failed to show HBsAg seropositivity in follow-up studies as OBI (7). Those who were HBV-DNA positive and exhibited HBsAg seroconversion during follow-up studies were defined as the window period (WP). Blood donors who were HBsAg positive in the supplementary serological assays were defined as having chronic HBV (CHB) infection.

### Analysis of inter-group similarity and variable correlation among blood donors

R language was used to compute the outcomes of 435 blood donations including the initial screening S/Co value, viral load, alternative PCR results, and HBV serological markers (HBsAg, anti-HBs, HBeAg, anti-HBe, and anti-HBc) for the principal co-ordinates analysis (PCoA) dimensionality reduction analysis. Permutational multivariate analysis of variance (PerMANOVA) was performed to determine whether there were significant differences between different groups including HBV infection and non-infection, and among three groups of HBV NAT yields. The generalized linear models (GLM) function was used to conduct univariate and multivariate analysis of basic clinical variables related to blood donors to verify the risk factors for HBV infection and to report the odds ratios (OR) with a 95% confidence interval (CI). Simultaneously, Spearman analysis was performed on the demographic data matrix of blood donors and the HBV supplementary experimental result matrix, and Mantel test analysis was conducted between the matrices to determine the correlation between variables of blood donors.

### Model construction and evaluation

**Figure 1:**
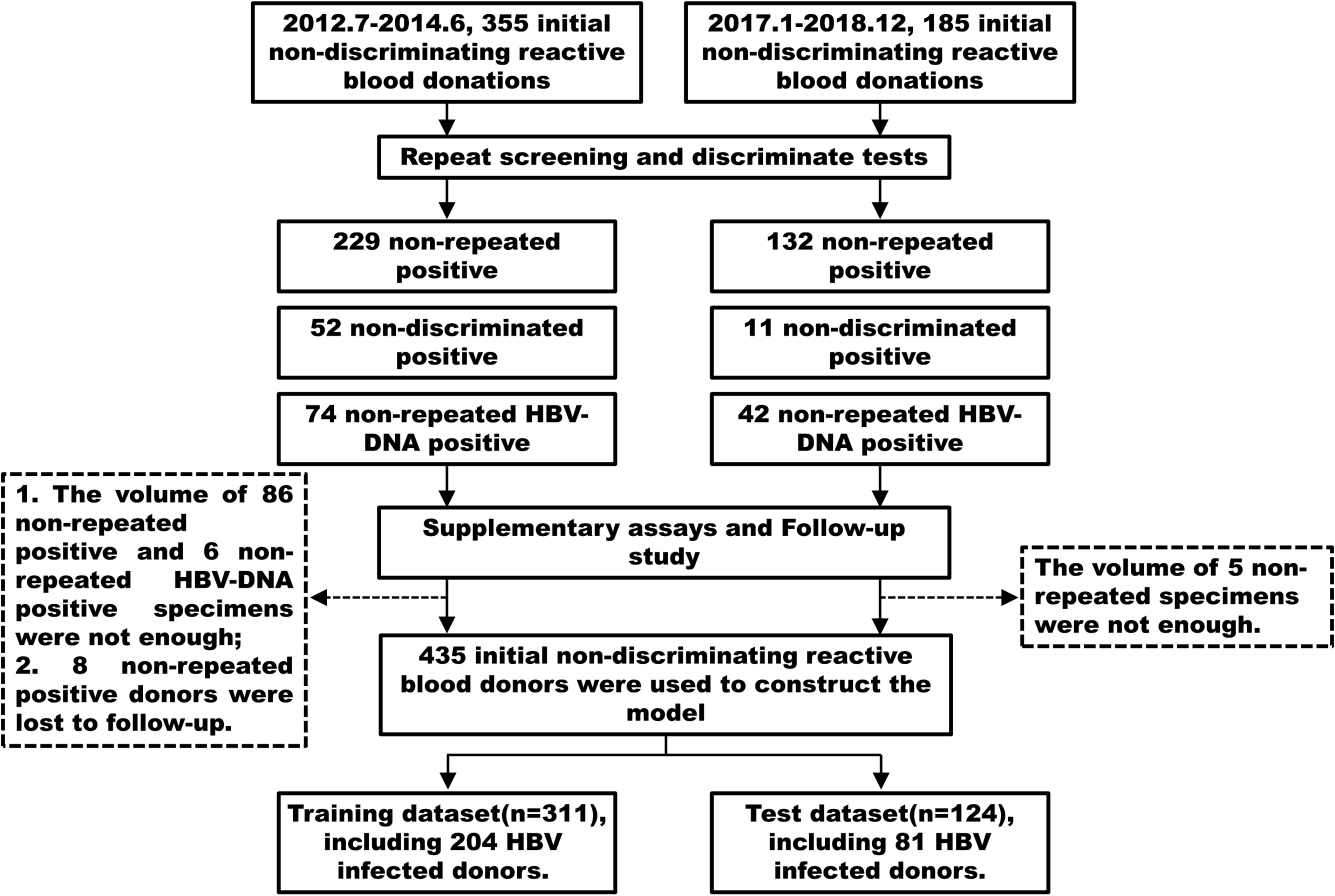
Number of donors enrolled and outcomes in the training and test datasets.

Through supplementary experiments and follow-up, we identified the HBV infection status of 435 initial non-discriminatory reactive blood donors and encoded it as a binary variable, with 1 indicating infection and 0 indicating non-infection. The 435 samples were randomly divided into a training dataset (311 cases) and a test dataset (124 cases) in a 7:3 ratio (Figure 1). The training dataset was used to develop a multivariate logistic regression prediction model, with the HBV infection status of the donors as the outcome. A stepwise regression algorithm was employed to identify the predictive factors of the model using the Akaike’s information criterion (AIC) as the stopping rule, selecting the model with the minimum AIC value and the fewest variables (12). Column line graphs were plotted based on the results of the multivariate logistic analysis.

Using nomograms, the HBV infection status of the blood donors in the validation dataset was calculated to validate and evaluate the model. The receiver operating characteristic (ROC) curve was plotted by comparing the training and validation datasets to calculate the area under the curve (AUC) as an assessment of the model’s discrimination. Generally, an AUC of 0.70–0.80 is considered moderate discrimination, while an AUC > 0.80 is considered high discrimination. The Hosmer–Lemeshow (H-L) test was used to evaluate the model’s goodness of fit, with a *p*-value >0.05, indicating a good fit and high calibration. The model’s effectiveness was evaluated through decision curve analysis (DCA), which analyzes the clinical decision curve. Finally, the model’s accuracy was determined by calculating the confusion matrix for the training and validation datasets.

### Data management and statistics

All data were processed using R 4.3.0 and STATA 17.0. The Mann–Whitney *U* test was used for continuous variables, and the Fisher exact χ^2^ test was used for categorical variables. All statistical tests were two-sided; *p* < 0.05 was considered statistically significant.

## Results

### HBV infection status of initial non-discriminatory reactive blood donors

A total of 435 cases of initial non-discriminatory reactive blood donors with HBV infection status were divided into three groups after two identical screening and discriminatory tests. Among them, 262 cases were in the non-repeat reactive group, 63 in the non-discriminatory reactive group, and 110 in the non-repeat HBV-DNA reactive group, indicating that the HBV discriminatory rate increased after repeated testing (110/435, 25.29%). Supplementary experiments and follow-ups were conducted on these 435 samples, including the ID-NAT model on different testing platforms, serological supplementary experiments, and viral load detection. The detection of HBV-DNA positive was defined as confirmed HBV infection. The results are presented in Table 1. There were four (1.53%), three (4.76%), and five (4.55%) cases of chronic HBV infection in the non-repeat reactive, non-discriminatory reactive, and non-repeat HBV-DNA reactive groups, respectively. No significant difference was observed among the groups (χ^2^ = 3.734, *p* = 0.15). Occult HBV infections comprised 116 (44.27%), 52 (82.54%), and 103 (93.64%) cases, respectively, with a significant difference among the groups (χ^2^ = 93.22, *p* < 0.01). It is worth noting that two window period cases were observed in the non-repeat HBV-DNA reactive group.

**Table 1:** The classification of HBV NAT yields according to alternative HBV NAT, serological markers, and follow-up studies in the different groups.

Groups of HBV NAT yields (total number)	Classification	Number (%)	Alt NAT^#^	HBV-DNA (VLs)	HBsAg^*^	Anti-HBs	Anti-HBc	Follow-up (HBV-DNA)
Non-repeated positive group (262)	Chronic infection	4 (1.53)	R	R/2NR	R	R/1NR	R	/
	OBI	24 (9.16)	R	R	NR	R	R	/
		16 (6.11)	R	R	NR	NR	R	/
		4 (1.53)	R	NR	NR	R/2NR	R/1NR^@^	/
		49 (18.70)	NR	NR	NR	R	R	R
		23 (8.78)	NR	NR	NR	NR	R	R
	Non-infection	59 (22.52)	NR	NR	NR	R	R	NR
		32 (12.21)	NR	NR	NR	NR	R	NR
		47 (17.94)	NR	NR	NR	R	NR	NR
		4 (1.53)	NR	NR	NR	NR	NR	NR
Non-discriminated positive group (63)	Chronic infection	3 (4.76)	R	R/1NR	R	R/2NR	R	/
	OBI	11 (17.46)	R	R	NR	R	R	/
		10 (15.87)	R	R	NR	NR	R	/
		4 (6.35)	R	NR	NR	R/2NR	R/3NR^@^	/
		13 (20.63)	NR	NR	NR	R	R	R
		14 (22.22)	NR	NR	NR	NR	R	R
	Non-infection	4 (6.35)	NR	NR	NR	R	NR	NR
		4 (6.35)	NR	NR	NR	NR	NR	NR
Non-repeated HBV-DNA positive group (110)	Chronic infection	5 (4.55)	R	R	R	NR	R	/
	Windows period	2 (1.82)	R	R	NR	NR	NR	R^&^
	OBI	19 (17.27)	R	R	NR	R	R	/
		26 (23.64)	R	R	NR	NR	R	/
		13 (11.82)	R	NR	NR	R/7NR	R/5NR^@^	/
		31 (28.18)	NR	NR	NR	R	R	R
		13 (11.82)	NR	NR	NR	NR	R	R
		1 (0.91)	NR	NR	NR	NR	NR	R

^#^Alternative NAT by using TaqMan PCR method; ^*^supplemental HBsAg test using CLIA method; ^@^anti-HBs single positive OBI; ^&^HBsAg seroconversion was observed during follow-up study; “R” means a positive result; “NR” means a negative result; VLs, viral loads; OBI, occult hepatitis B virus infection; %, percentage in group total number.

**Figure 2:**
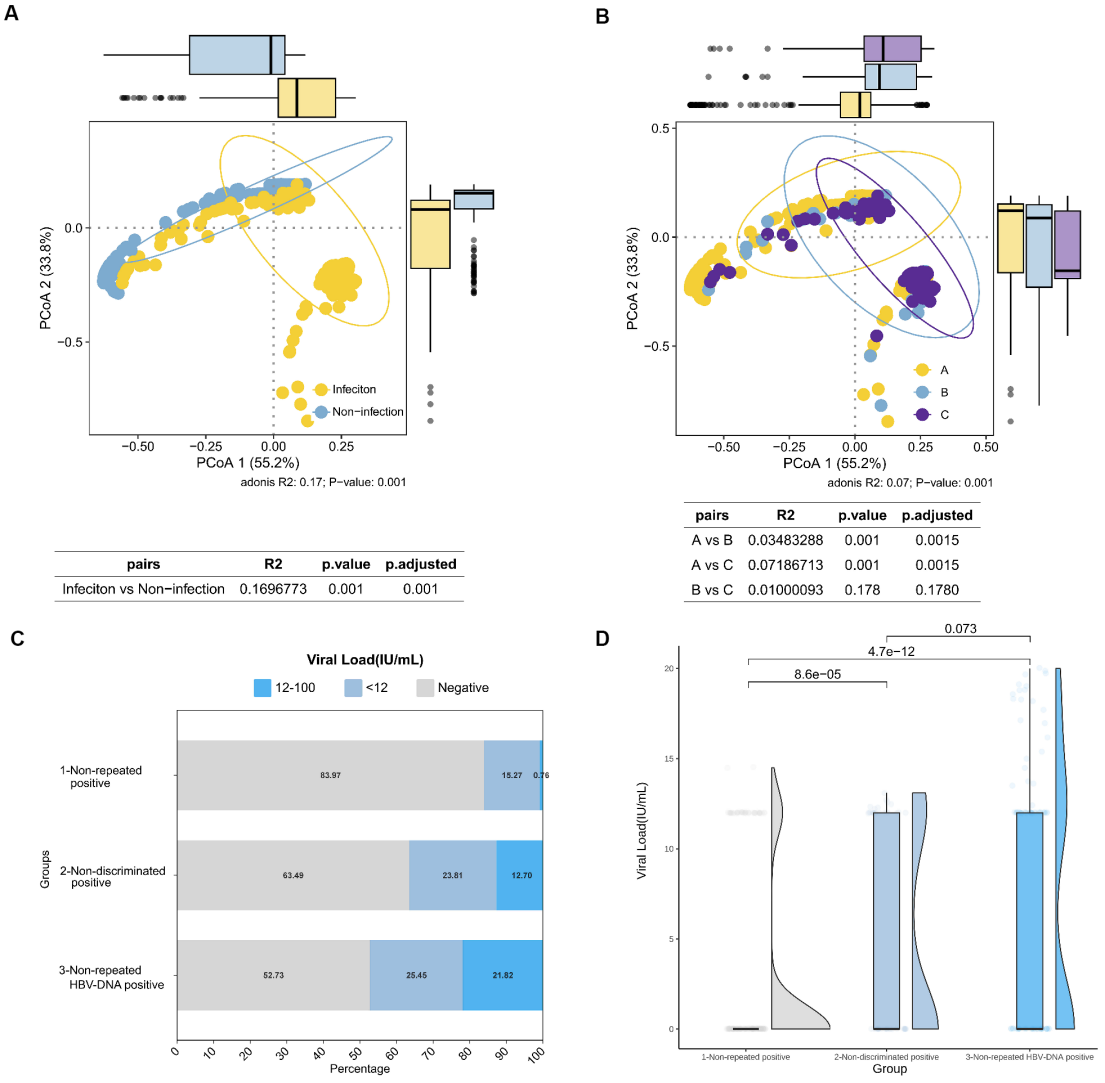
Comparison of HBV infection status among different groups of HBV NAT yields blood donors. **(A)** PCoA results in HBV-infected and non-infected states. **(B)** PCoA results among different groups of HBV NAT yields, where A, B, and C represent the non-repeated positive group, non-discriminated positive group, and non-repeated HBV-DNA positive group, respectively. **(C)** Proportional distribution of viral load among negative, <12 IU/mL, and 12–100 IU/mL in different groups of HBV NAT yields. **(D)** Comparison of viral loads among different groups of HBV NAT yields.

The results of the PCoA dimensionality reduction analysis were conducted on the initial screening S/Co values, viral load, PCR results, and serological markers (HBsAg, anti-HBs, HBeAg, anti-HBe, and anti-HBc) of 435 blood donors. PerMANOVA, alternatively known as ADONIS analysis, was performed to compare the differences between HBV infected and non-infected individuals, and among three HBV NAT yields groups. The analysis revealed significant differences among all groups. The PerMANOVA test based on the Bray-Curtis distance measures demonstrated that the difference between HBV infected and non-infected donors was significant (*p* = 0.001; Figure 2A), and also among the three groups of HBV NAT yields (*p* = 0.001; Figure 2B). However, there was no significant difference between the non-discriminated positive and the non-repeated HBV-DNA positive groups (*p* = 0.178; Figure 2B). This implies that if a sample tests positive in repeated testing, it is highly possibly in a state of HBV infection, regardless of whether repeated screening or discriminatory experiments are used.

Viral loads varied among the different groups. In the non-repeated reactive group, the majority (83.97%) had a negative viral load, whereas in the HBV-discriminated positive group, only 52.73% had a negative viral load, and the proportion of viral load >12 IU/mL was the highest (21.82%; Figure 2C). When comparing the viral load values among the three groups, the non-repeated positive group had a lower viral load than the other two groups (*p* < 0.01; Figure 2D). However, the non-discriminated positive group and the non-repeated HBV-DNA positive group did not differ significantly, which is consistent with the results of PCoA, indicating no significant difference in HBV viral load and serological markers between these two groups. These outcomes indicate that the HBV serological markers and viral load in the non-repeated positive group were different from those in the other two groups, with a lower viral load and a higher proportion of false positives.

### Comparison of basic clinical variables of initial non-discriminatory reactive blood donors

Among the 435 blood donors included in the study, 150 were found to be uninfected with HBV, and 285 were HBV-infected, with the majority being OBI (Table 1), resulting in an infection rate of 65.52% (285/435). The basic clinical variables recorded for the blood donors included the initial screening S/Co value, screening reagents, and repeat experiments, and demographic data, including age, gender, native place, hepatitis B vaccination status after birth, ABO and RhD blood types, body mass index, marital status, education level, occupation, blood donation type, interval time of donation (days), times of donation and types of donors. When comparing the proportion differences between HBV-infected and non-infected blood donors for different variables, significant differences were observed in the initial screening S/Co values, screening reagents, and repeat experiments among the blood donor samples. Among the demographic data of the blood donor population, significant differences were found in terms of native place, age, marital status, and education level (Table 2).

**Table 2:** Basic clinical characteristics between HBV infected and non-infected blood donors who were initial non-discriminatory reactive.

Basic clinical characteristics^@^		HBV Non-infection (*n* = 150)	HBV infection (*n* = 285)	*p-*value^#^
S/Co value of Screening Test, $\bar{x}$ ± s		10.1 ± 5.2	12.7 ± 3.9	<0.01
Screening reagents, *n* (%)				<0.01
Procleix^®^ Ultrio^®^ Assay		73 (48.7)	182 (63.9)	
Procleix^®^ Ultrio Elite^®^ Assay		77 (51.3)	103 (36.1)	
Groups of HBV NAT yields, *n* (%)				<0.01
Non-repeated positive group		142 (94.7)	120 (42.1)	
Non-discriminated positive group		8 (5.3)	55 (19.3)	
Non-repeated HBV-DNA positive group		0 (0.0)	110 (38.6)	
Native place, *n* (%)				0.03
Higher prevalence areas	Zhejiang	99 (66.0)	223 (78.2)	
	Fujian	2 (1.3)	1 (0.4)	
	Jiangxi	4 (2.7)	15 (5.3)	
	Hubei	4 (2.7)	5 (1.8)	
	Hunan	1 (0.7)	1 (0.4)	
	Guangxi	0 (0.0)	1 (0.4)	
	Sichuan	5 (3.3)	7 (2.5)	
	Guizhou	0 (0.0)	1 (0.4)	
Lower prevalence areas^*^	Anhui	18 (12.0)	16 (5.6)	
	Hebei	1 (0.7)	0 (0.0)	
	Liaoning	1 (0.7)	0 (0.0)	
	Jilin	1 (0.7)	0 (0.0)	
	Heilongjiang	1 (0.7)	1 (0.4)	
	Shandong	2 (1.3)	0 (0.0)	
	Shanxi	1 (0.7)	0 (0.0)	
	Henan	8 (5.3)	10 (3.5)	
	Jiangsu	2 (1.3)	3 (1.1)	
	Shanghai	0 (0.0)	1 (0.4)	
Age, M (Q1, Q3)		39 (31, 47)	42 (37, 48)	<0.01
Vaccination status at birth, *n* (%)				0.77
Non-vaccination		145 (96.7)	277 (97.2)	
Vaccination		5 (3.3)	8 (2.8)	
Gender, *n* (%)				0.16
Female		43 (28.7)	64 (22.5)	
Male		107 (71.3)	221 (77.5)	
ABO blood type, *n* (%)				0.32
A		41 (27.3)	105 (36.8)	
B		46 (30.7)	69 (24.2)	
O		52 (34.7)	91 (31.9)	
AB		11 (7.3)	20 (7.0)	
RhD blood type, *n* (%)				0.55
RhD^−^		0 (0.0)	3 (1.1)	
RhD^+^		150 (100.0)	282 (98.9)	
BMI, $\bar{x}$ ± s		23.9 ± 2.1	24.2 ± 1.8	0.77
Marital Status, *n* (%)				
Unmarried		73 (48.7)	33 (11.6)	<0.01
Married		67 (44.7)	175 (61.4)	
Other		10 (6.7)	77 (27.0)	
Education level, *n* (%)				
Primary school		1 (0.7)	6 (2.1)	0.03
Junior high school		40 (26.7)	64 (22.5)	
Middle school		46 (30.7)	72 (25.3)	
College		16 (10.7)	38 (13.3)	
Undergraduate		24 (16.0)	29 (10.2)	
Graduate and above		2 (1.3)	2 (0.7)	
Other		21 (14.0)	74 (26.0)	
Occupation, *n* (%)				0.14
Farmer		19 (12.7)	63 (22.1)	
Worker		16 (10.7)	35 (12.3)	
Self-employed		17 (11.3)	38 (13.3)	
Clerk		31 (20.7)	41 (14.4)	
Military		1 (0.7)	1 (0.4)	
Student		4 (2.7)	6 (2.1)	
Medical staff		0 (0.0)	4 (1.4)	
Government employee		3 (2.0)	6 (2.1)	
Other		59 (39.3)	91 (31.9)	
Donation type, *n* (%)				0.42
Whole blood		116 (77.3)	210 (73.7)	
Platelet		34 (22.7)	75 (26.3)	
Interval time of donation (days), M (Q1, Q3)		434 (257, 3,650)	455 (189, 3,650)	0.43
Times of donation, M (Q1, Q3)		3 (2, 7)	3 (1, 11)	0.50
Types of donors, *n* (%)				0.41
Non-regular blood donors		98 (65.3)	174 (61.1)	
Regular blood donors		52 (34.7)	111 (38.9)	

^@^The normal distribution of numerical variables is represented by the mean ± standard deviation ( $\bar{x}$ ± s), non-normal distributions are represented by the median [first quartile, third quartile; M (Q1, Q3)], and categorical variables are represented by frequency [percentage; *n* (%)]. ^*^Compared with the higher prevalence areas of HBV, there is a significant difference (*p* < 0.01; Fisher’s exact), and the determination of high and low prevalence areas based on previous research (13). ^#^Bivariate analyses were conducted using either the Fisher exact test or the Wilcoxon rank-sum test; the *p*-value was derived from bivariate association analyses between each study variable and the HBV infection state.

**Figure 3:**
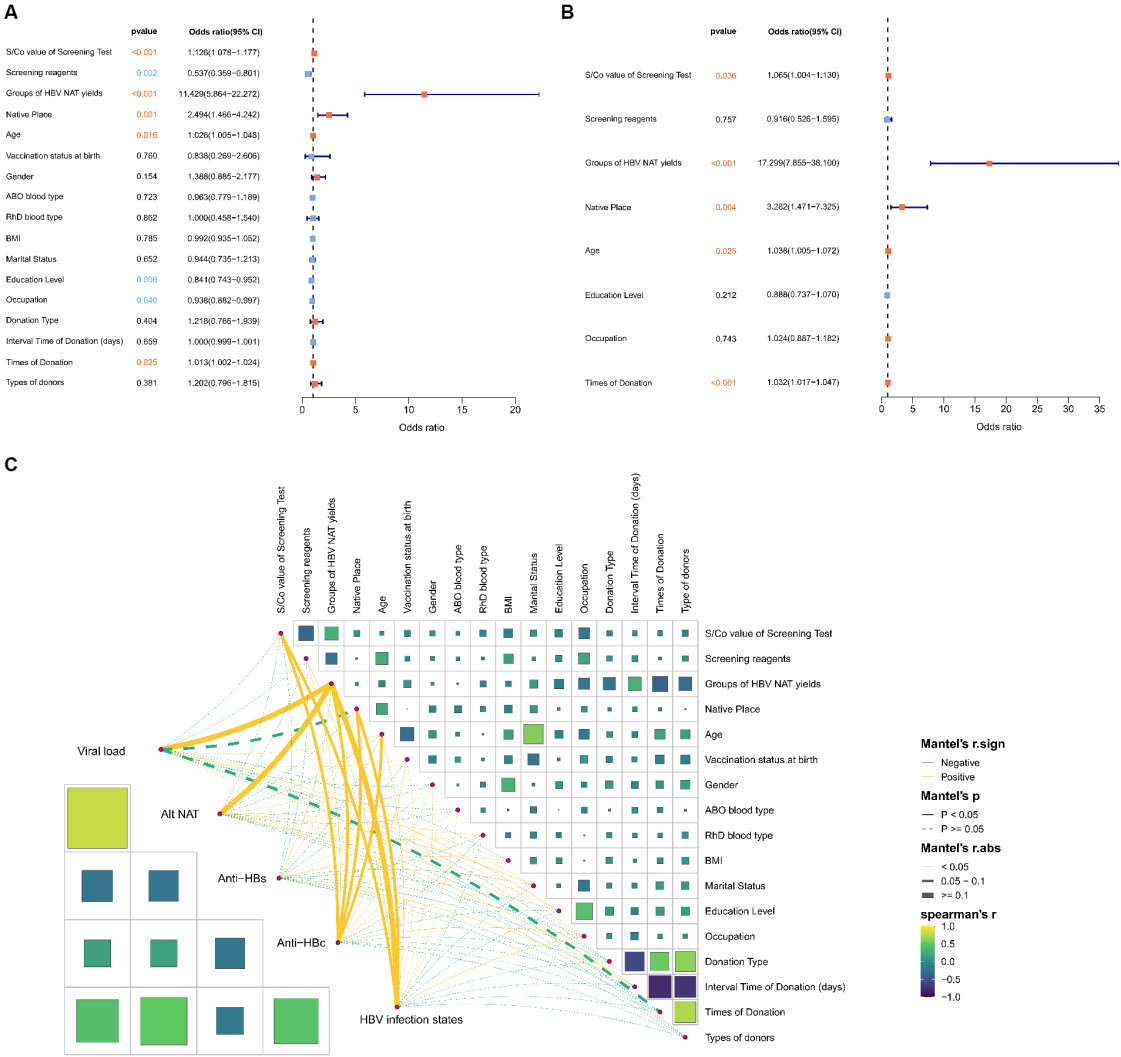
Correlation between basic clinical variables and HBV infection status of initial non-discriminatory reactive blood donors. Univariate **(A)** and multivariate **(B)** analyses of basic clinical variables related to blood donors to determine the risk factors for HBV infection. **(C)** Spearman analysis results on two matrices to determine the correlation between variables of blood donors, including the basic variable data matrix and the HBV supplementary experimental results matrix; the Mantel test analysis results between the matrices to determine the correlation between the matrices.

Univariate analysis was performed on all variables demonstrated statistically significant differences in the following seven variables: initial S/Co value of the screening test, screening reagents, groups of HBV NAT yields, age, marital status, education level, and times of donation (Figure 3A). Multivariate analysis revealed a high S/Co value, non-repeated HBV-DNA positive group, older age, and more donations, which are risk factors for HBV infection (Figure 3B).

Moreover, using the Mantel test, matrix analysis was conducted on the basic clinical data of the blood donor population and HBV infection data to explore the correlation between viral load, alt-NAT, anti-HBs, anti-HBc, HBV infection status, and basic clinical factors (Figure 3C). The initial S/Co value of the screening test, groups of HBV NAT yields, and native place were significantly positively correlated with HBV infection (*r* ≥ 0.05, *p* < 0.05), indicating that higher initial screening values, positive in repeat tests, and blood donors from high prevalence areas were positively correlated with HBV infection, consistent with the aforementioned multivariate results. The presence of anti-HBc was significantly correlated with HBV infection status. Furthermore, the positive correlation with factors related to HBV infection mentioned above, anti-HBc also significantly correlates with age (*r* ≥ 0.05, *p* < 0.05), a risk factor for HBV infection.

### Construction of predictive model for HBV infection status in initial non-discriminatory reactive blood donors

**Figure 4:**
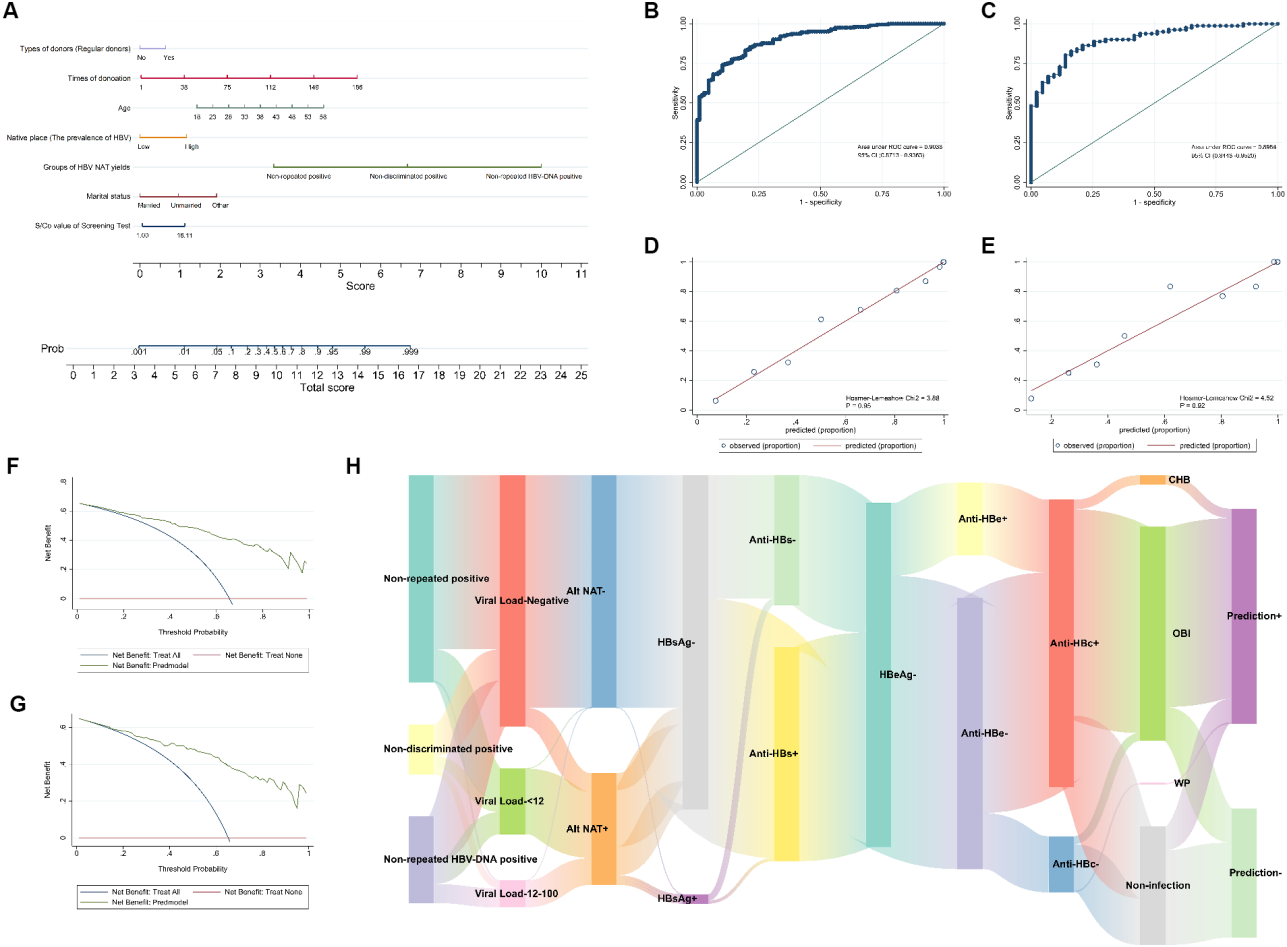
A predictive model for the hepatitis B infection status of initial non-discriminatory reactive blood donors. **(A)** Nomogram to estimate the probability of HBV infection status in blood donors with non-discriminatory reactive results. The ROC curves of the nomograms in the training **(B)** and test **(C)** datasets. The calibration curve of nomogram for predicting HBV infection status in the training **(D)** and test **(E)** datasets. The DCA of training **(F)** and test **(G)** datasets. **(H)** The Sankey diagram depicts the final predicted outcomes of non-discriminatory reactive blood donors in different groups of HBV NAT yields.

Data from 311 donors were used to establish the nomogram predictive model, and data from 124 donors were used to evaluate its performance (Table 3). The proportion of HBV infection in the training and test datasets was 48.6 and 47.6%, respectively. No statistically significant differences were observed between the training and test datasets for the basic clinical variables examined (Table 3). After variable selection using the multivariable regression model, the S/Co value of the screening test, marital status, groups of HBV NAT yields, native place, age, times of donation, and types of donors were selected as the best subset of predictors for the probability of HBV infection (Table 4). The nomogram incorporating these predictors is illustrated in Figure 4A. The nomogram had good discriminative power with an area under the ROC curve of 0.90 (95% CI: 0.87–0.94) and 0.90 (95% CI, 0.84–0.95) in the training and test datasets, respectively (Figures 4B,C). Moreover, the nomogram was well calibrated with a Hosmer-Lemeshow χ^2^ statistic of 3.88 (*p* = 0.95) and 4.52 (*p* = 0.92) in the training and test datasets, respectively (Figures 4D,E). The DCA of training and test datasets indicated that the model can effectively predict the HBV infection status of blood donors (Figures 4F,G). The confusion matrices for the training and test datasets are presented in Table 5. The accuracy (number of correct predictions divided by the number of total predictions) was 82.96 and 83.06% in the training and test datasets, respectively (Table 5). The Sankey diagram indicated that CHB, WP, and most of OBIs could be correctly predicted as HBV infections; however, 10.11% (44/435) of the total OBIs were predicted to be non-infectious (Figure 4H).

**Table 3:** Summary of study variables stratified by datasets.

Variable^@^		Training dataset (*n* = 311)	Test dataset (*n* = 124)	*p-*value^#^
S/Co value of Screening Test, $\bar{x}$ ± s		11.7 ± 4.6	12.0 ± 4.6	0.56
Screening reagents, *n* (%)				0.39
Procleix^®^ Ultrio^®^ Assay		178 (57.2)	77 (62.1)	
Procleix^®^ Ultrio Elite^®^ Assay		133 (42.8)	47 (37.9)	
Groups of HBV NAT yields, *n* (%)				0.82
Non-repeated positive group		189 (60.8)	73 (58.9)	
Non-discriminated positive group		43 (13.8)	20 (16.1)	
Non-repeated HBV-DNA positive group		79 (25.4)	31 (25.0)	
Native place, *n* (%)				0.39
Higher prevalence areas	Zhejiang	227 (73.0)	95 (76.6)	
	Fujian	3 (1.0)	0 (0.0)	
	Jiangxi	12 (3.9)	7 (5.6)	
	Hubei	7 (2.3)	2 (1.6)	
	Hunan	2 (0.6)	0 (0.0)	
	Guangxi	0 (0.0)	1 (0.8)	
	Sichuan	6 (1.9)	6 (4.8)	
	Guizhou	1 (0.3)	0 (0.0)	
Lower prevalence areas^*^	Anhui	27 (8.7)	7 (5.6)	
	Hebei	1 (0.3)	0 (0.0)	
	Liaoning	0 (0.0)	1 (0.8)	
	Jilin	0 (0.0)	1 (0.8)	
	Heilongjiang	2 (0.6)	0 (0.0)	
	Shandong	2 (0.6)	0 (0.0)	
	Shanxi	1 (0.3)	0 (0.0)	
	Henan	15 (4.8)	3 (2.4)	
	Jiangsu	4 (1.3)	1 (0.8)	
	Shanghai	1 (0.3)	0 (0.0)	
Age, M (Q1, Q3)		42(34, 47)	42 (35, 48)	0.73
Vaccination status at birth, *n* (%)				1.00
Non-vaccination		302 (97.1)	120 (96.8)	
Vaccination		9 (2.9)	4 (3.2)	
Gender, *n* (%)				0.14
Female		83 (26.7)	24 (19.4)	
Male		228 (73.3)	100 (80.6)	
ABO blood type, *n* (%)				0.52
A		110 (35.4)	36 (29.0)	
B		82 (26.4)	33 (26.6)	
O		99 (31.8)	44 (35.5)	
AB		20 (6.4)	11 (8.9)	
RhD blood type, *n* (%)				0.20
RhD^−^		1 (0.3)	2 (1.6)	
RhD^+^		310 (99.7)	122 (98.4)	
BMI, $\bar{x}$ ± s		24.4 ± 3.5	24.1 ± 3.0	0.63
Marital Status, *n* (%)				0.61
Unmarried		74 (23.8%)	32 (25.8%)	
Married		171 (55.0%)	71 (57.3%)	
Other		66 (21.2%)	21 (16.9%)	
Education Level, *n* (%)				0.92
Primary school		4 (1.3)	3 (2.4)	
Junior high school		74 (23.8)	30 (24.2)	
Middle school		87 (28.0)	31 (25.0)	
College		39 (12.5)	15 (12.1)	
Undergraduate		35 (11.3)	18 (14.5)	
Graduate and above		3 (1.0)	1 (0.8)	
Other		69 (22.2)	26 (21.0)	
Occupation, *n* (%)				0.38
Farmer		54 (17.4)	28 (22.6)	
Worker		38 (12.2)	13 (10.5)	
Self-employed		41 (13.2)	14 (11.3)	
Clerk		48 (15.4)	24 (19.4)	
Military		0 (0.0%)	2 (1.6%)	
Student		8 (2.6%)	2 (1.6%)	
Medical staff		3 (1.0%)	1 (0.8%)	
Government employee		6 (1.9%)	3 (2.4%)	
Other		113 (36.3)	37(29.8)	
Donation type, *n* (%)				0.54
Whole blood		230 (74.0)	96 (77.4)	
Platelet		81 (26.0)	28 (22.6)	
Interval time of donation (days), M (Q1, Q3)		455.0 (207.0, 3650.0)	431.0 (194.5, 3650.0)	0.66
Times of Donation, M (Q1, Q3)		3.0 (1.0, 9.0)	3.0 (1.0, 7.5)	0.46
Types of donors, *n* (%)				0.83
Non-regular blood donors		193 (62.1)	79 (63.7)	
Regular blood donors		118 (37.9)	45 (36.3)	
HBV infection states, *n* (%)				1.00
HBV non-infection		107(34.4)	43 (34.7)	
HBV infection		204(65.6)	81 (65.3)	

^@^The normal distribution of numerical variables is represented by the mean ± standard deviation ( $\bar{x}$ ± s), non-normal distributions are represented by the median [first quartile, third quartile; M (Q1, Q3)] and categorical variables are represented by frequency [percentage; *n* (%)]. ^*^Compared with the higher prevalence areas of HBV, there is no significant difference (*p* > 0.05; Fisher’s exact), and the determination of high and low prevalence areas based on previous research (13). ^#^Bivariate analyses were conducted using either the Fisher exact test or the Wilcoxon rank-sum test; the *p*-value was derived from bivariate association analyses between each study variables and the HBV infection state.

**Table 4:** Predictors for the Hepatitis B infection in initial non-discriminated reactive blood donors in the final regression model for the training dataset.

Intercept and variable	β Coefficient	Odds ratio (95% CI)	*P-*value
S/Co value of Screening Test	0.0615309	1.065(0.988–1.147)	0.099
Marital Status	−0.9378324	0.414(0.259–0.660)	0.000
Groups of HBV NAT yields	3.563502	32.734(11.332–94.557)	0.000
Native place	1.382004	4.074(1.577–10.526)	0.004
Age	0.0780915	1.076(1.030–1.125)	0.001
Times of donation	0.0273489	1.027(1.009–1.045)	0.003
Types of donors	0.8111636	2.141(1.005–4.562)	0.048
Intercept	−8.504637	NA	NA

CI, confidence interval; NA, not applicable.

**Table 5:** Summary of 2 × 2 table of the confusion matrix for training and test datasets.

Predicted outcome	Actual outcome		The accuracy
	HBV infection	HBV non-infection	
Training dataset			
HBV infection	174	23	82.96%
HBV non-infection	30	84	
Test dataset			
HBV infection	67	7	83.06%
HBV non-infection	14	36	

### Comparison of screening strategies for initial non-discriminatory reactive samples

According to the PCoA results (Figure 2B), there was no significant difference between the non-discriminated positive and non-repeated HBV-DNA positive groups, concluding that repeated screening tests have little impact on the classification of HBV-infected populations; therefore, the number of repeated screening tests can be reduced. We performed an approximate cost-effectiveness analysis by comparing the following five strategies: (i) two repeat discriminate tests + re-entry tests, (ii) two repeat screening tests + two repeat discriminate tests + re-entry tests, (iii) two repeat discriminate tests + model predictions + re-entry tests, (iv) two repeat screening tests + two repeat discriminate tests + model predictions + re-entry tests, and (v) no repeat tests + re-entry tests (Table 6). Strategy (iii), two repeat discriminate tests + model predictions + re-entry tests, was the most highly cost-effective. The program also aimed to allow as many non-repeated positive blood donors as possible to return to the team (164/262, 62.60%), ensuring the blood donor population. However, this model resulted in 15.49% (22/142) of NAT false positive blood donors being classified as positive in non-repeated positive groups, thus preventing them from returning to the team. It was estimated to affect approximately 283 blood donors, accounting for only 87.94 per million (283/3218194) of the total number of blood donors.

**Table 6:** Cost-effectiveness analysis of 3,218,194 donors^*^ in the Blood Center of Zhejiang Province.

Cost (RMB)	Screening strategy														
	Two repeat discriminate tests + re-entry tests			Two repeat screening tests + two repeat discriminate tests + re-entry tests			Two repeat discriminate tests + model predictions + re-entry tests			Two repeat screening tests + two repeat discriminate tests + model predictions + re-entry tests			Benefit (re-entry tests) (RMB)		
	Donor number	Per test cost^@^	Total cost	Donor number	Per test cost	Total cost	Donor number	Per test cost	Total cost	Donor number	Per test cost	Total cost	Donor number	Per test cost	Total cost
Repeat tests	/	/	/	5,927	59.50	705251.72	/	/	/	5,927	59.50	705251.72	/	/	/
Repeat discriminate tests	5,927^&^	59.50	705251.72	5,927	59.50	705251.72	5,927	59.50	705251.72	5,927	59.50	705251.72	/	/	/
Re-entry tests	4,890	1228.20	6005414.56	4,537	1228.20	5572803.05	3,061^#^	1228.20	3759114.46	2,840^#^	1228.20	3488319.47	5,927	1228.20	7279414.94
total tests	6710666.28			6983306.50			4464366.18			4898822.92			7279414.94		
Benefit/cost	1.08			1.04			1.63			1.49			1.00		

^*^Between August 1, 2010, and December 31, 2022, a total of 3,218,194 donations were screened by nucleic acid testing in the Blood Center of Zhejiang Province. ^&^Non-repeated positive, non-discriminated positive and non-repeated HBV-DNA positive NAT yields (per million) in Zhejiang Province were 1409.91, 109.45 and 322.32, respectively (8). ^#^The prediction model for predicting the HBV infection status rates in different groups of HBV NAT yields are 100% in the non-repeated HBV-DNA positive group, 100% in the non-discriminated positive group and 37.40% in the non-repeated positive group. ^@^Repeat screening and discriminate test: Grifols NAT for HBV-DNA/HCV-RNA/HIV-RNA/ID format, 59.5 RMB/test. The reagents for re-entry tests include the following several types: Bio-Rad MONOLISA HBsAg ELISA, 6.82 RMB/test; InTec ADVANCED Diagnostic Kit for HBsAg ELISA, 0.66 RMB/test; Grifols NAT for HBV-DNA/HCV-RNA/HIV-RNA/ID format, 59.5 RMB/test; Roche MPX2.0 NAT for HBV-DNA/HCV-RNA/HIV-RNA/ID format, 60.4 RMB/test; Abbott Diagnostics CLIA for HBsAg, anti-HBs, HBeAg, anti-HBe, anti-HBc and other cost, 1100.82 RMB/test. 1$ = 7.1RMB.

**Figure 5:**
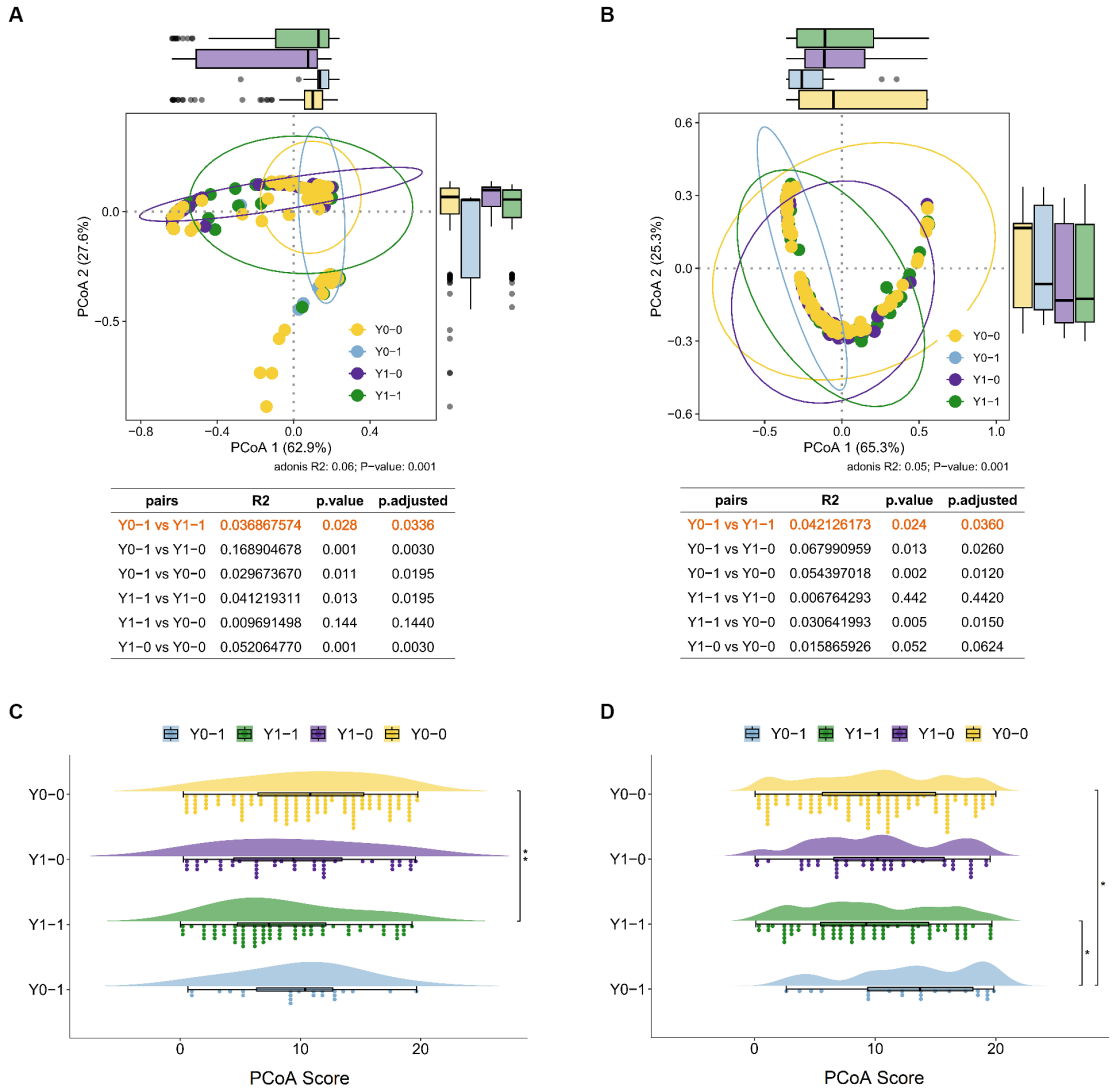
The PCoA of characteristics of non-repeat reactive blood donors. PCoA of HBV infection characteristics **(A)** and basic clinical characteristics **(B)** in four groups, including predicted positive but actually negative (Y0-1) and positive (Y1-1) blood donors, and predicted negative but actually negative (Y0-0) and positive (Y0-1) blood donors. The comparison of PCoA score of HBV infection characteristics **(C)** and basic clinical characteristics **(D)** in four groups using the Wilcoxon rank-sum test. ^*^p < 0.05, ^**^p < 0.01.

The PCoA of HBV infection characteristics of these non-repeat reactive blood donors revealed differences among the four different prediction results (*p* = 0.001), and there was a significant difference between predicted positive but actually negative (Y0-1) and positive (Y1-1) blood donors (*p* = 0.028; Figure 5A). Nevertheless, the calculated PCoA score comparisons showed no significant differences between the two groups (*p* = 0.25; Figure 5C). Moreover, the PCoA of the basic clinical characteristics of these non-repeat reactive blood donors indicated differences among the four different prediction results (*p* = 0.001), particularly between the predicted positive but actually negative (Y0-1) and positive (Y1-1) blood donors (*p* = 0.024; Figure 5B). Comparisons of the calculated PCoA scores also presented significant differences between the two groups (*p* = 0.017; Figure 5D). Therefore, for the blood donors identified as true positives by the predictive model, PCoA calculations of basic clinical characteristics were conducted to determine those with a value greater than 10, and they were included in the final selection, accounting for 53.06% of the total positive cases. According to the calculations, strategies (iii) and (iv) would increase the cost by approximately 1,191,914.3 RMB and 1,106,052.5 RMB, respectively. The benefit/cost ratios for strategy (iii) and strategy (iv) were 1.29 and 1.21, respectively. Accordingly, strategy (iii) remains the most effective among the four screening strategies. Therefore, combining the results of the two identification experiments and the predictive model can maximize the benefit/cost ratio and reduce resource wastage.

## Discussion

In recent decades, substantial developments have been made in donor selection, screening strategies, efficient serological and molecular detection analyses, and the implementation of pathogen reduction technologies in blood components, significantly improving viral blood safety. Blood screening plays a crucial role in reducing the transmission of infectious diseases through blood transfusions. In terms of public awareness, particularly among all stakeholders involved in blood transfusions, transfusion safety related to infectious diseases remains an extremely sensitive subject. Consequently, any positive screening result, even with doubts, can prompt a decision to abandon donation and permanently defer donation to avoid risks and ensure optimal transfusion safety. However, improved analytical sensitivity and increased targeted viral markers using detection methods elevate the risk of false-positive outcomes (14, 15). Amidst a widespread blood shortage, the discarding of blood donations and permanent deferral of donors due to false-positive blood test results has garnered heightened attention (9–11). Therefore, the final interpretation of positive screening results can be highly challenging for TMA screening methods, where initially reactive donations in multiplex testing may yield in non-reactive results in discriminatory analysis. Studies have reported that these donors expected to have a Poisson distribution of HBV-DNA levels near the limit of detection (LOD) in the blood, particularly in OBI donors characterized by extremely low HBV-DNA levels in the plasma (2). Our research also found that these donors experience fluctuations in viral load, significantly affecting the detection results (8). This phenomenon requires blood screening laboratory personnel to frequently confront the challenge of classifying an isolated reactive result as true positive or false positive.

In this study, the anti-HBc positivity rate in the initial screening of non-repeat reactive blood donors was 80.15% (210/262) in the non-repeat reactive group, 82.54% (52/63) in the non-discriminatory reactive group, and 92.73% (102/110) in the non-repeat HBV-DNA reactive group, with a total of 83.68% (364/435), which is consistent with our previous research results of 83.57% (229/274) (8). Similar findings were observed in studies conducted in Chinese blood centers, including 91.1% in Shenzhen (16) and 87.3% (69/79) in Dalian (17). However, these rates are higher than those reported in Korea (47%) (18) and New Zealand (13–57%) (6). In China the anti-HBc positivity rates in non-discriminatory reactive donors are higher than those in qualified blood donors (47.4%) (19). Our study observed a significant correlation between anti-HBc and HBV infection status (cor = 0.45, *p* < 0.05), indicating that anti-HBc testing can ensure blood safety by identifying occult HBV infections that cannot be detected by NAT, which was also reported by the Taormina Workshop on Occult HBV Infection (7). However, there are limitations in conducting anti-HBc screening for blood donors in China. The rate of anti-HBc positivity in China’s general population has been reported as 30–40% (20, 21). Therefore, the routine screening for anti-HBc will result in a deferral of a significant number of blood donors. Furthermore, anti-HBc screening cannot identify WP infection and has low specificity, with false positive rates ranging from 16 to 75% (22). In addition, studies have also found a certain proportion of OBI blood donors who are anti-HBs positive but anti-HBc non-reactive (2, 16, 23, 24). In this study, we also identified 6 cases of OBI blood donors who were anti-HBs single reactive. These cases have been reported to be associated with transfusion-transmitted infections (25). A study reported that adding anti-HBc screening for MP+/ID-non-resolved donations can effectively reduce HBV transfusion transmission and minimize the elimination rate of blood donors, with optimal cost-effectiveness (26). Therefore, in China, HBc screening can only serve as a supplementary test rather than a screening method.

Developing a simpler and more feasible screening strategy is easier to implement, whereas the method of repeated testing is more viable. In this study, this method increased the detection rate of HBV-DNA by 25.29% (110/435), and all were confirmed as HBV-infected individuals, of which 93.64% (103/110) were OBI, 1.82% (2/110) were WP, and 4.55% (5/110) were low concentration HBsAg CHB. Similarly, a proportion of 14.48% (63/435) tested positive in the repeated screening experiments, with 87.30% (55/63) being HBV-infected, of which 82.54% (52/63) were OBI and 4.76% (3/63) were CHB. Non-repetitive reactive blood donors accounted for 60.23% (262/435), of which 44.27% (116/262) were OBI and 1.53% (4/262) were low concentration HBsAg CHB. There was a significant positive correlation (cor = 0.17, *p* < 0.05) between viral load and HBV NAT yield groups, indicating that the higher the viral load, the higher the probability of being identified or tested positive. PCoA of HBV infection markers among different detection groups revealed that blood donors who tested positive in repeated screening or discriminatory experiments were more likely to be HBV-infected. However, there was no significant difference between the non-discriminated positive and non-repeated HBV-DNA positive groups (Figure 2B). Consequently, repeated testing can somewhat distinguish the HBV infection status, but the type of repeated experiment can be selected independently. Similar repeated experiments were also conducted at the Dalian Blood Center to determine the infection status of blood donors, but they were more stringent (17). Virions were concentrated from 12 and 6 mL plasma samples by ultracentrifugation (UC) and polyethylene glycol (PEG) precipitation, respectively. HBV-DNA was detected using four nested polymerase chain reactions (95% LOD: 5–25 IU/mL). Amplified products were sequenced for definitive confirmation, revealing that 43.04% (34/79) to 69.62% (55/79) were HBV-DNA positive (17). At the Shenzhen Blood Center, ultracentrifugation of large volumes of plasma samples was performed and tested by nested PCR or alternative qPCR, indicating 45.95% (119/259) were HBV-DNA positive (16). In this experiment, the blood donors who exhibited reactive results in repeated experiments accounted for 39.77% (173/435), although lower than those in Shenzhen and Dalian, it was easier to execute and required fewer samples, rendering it more practical.

In our study, 45.80% (120/262) of non-repeat reactive blood donors demonstrated a certain degree of HBV infection, and all tested negative in repeat testing. Additional experiments, including viral load detection, HBV serological markers, and follow-up experiments (2, 16, 17, 27–29), were conducted to determine the infection status of the blood donors. However, research has proven that the confirmation rate of follow-up is considerably lower than that of confirmation/supplementary experiments, due to the compliance of blood donors with follow-up and the fluctuation of blood-borne viruses (2). To improve the predictability of their infection status, we innovatively constructed a predictive model by selecting over 10 variables from routine screening and recorded blood donor data. The model included the following seven variables: the S/Co value of the screening test, marital status, groups of HBV NAT yields, native place, age, times of donation, and types of donors. The model’s discrimination, goodness of fit, effectiveness, and accuracy were evaluated through the analysis of indicators such as AUC, DCA, and confusion matrix, and it was found that the model had a good predictive performance. This study had certain limitations. The model’s predictive data in this study were obtained from a small sample from a single center in Zhejiang Province. Although internal validation and model performance evaluation yielded positive results, whether it can be extrapolated to other populations remains uncertain. Moreover, further external validation from multiple centers is necessary.

Currently, to reduce the loss of blood donors, alleviate the shortage of blood supply, and resolve disputes between blood banks and blood donors, some blood banks have implemented a strategy of returning blood donors to teams in China (2, 30–36). The primary objective is to identify suspected false-positive results so that potential blood donors can re-entry. It is stipulated that only blood donors with non-discriminatory NAT reactivity can be re-entry to the team if they test positive for nucleic acids in Zhejiang Province (34–36). Therefore, it is necessary to identify and determine the infection status of blood donors by using discriminatory tests in the screening strategy. Testing for blood donor re-entry includes routine screening and confirmatory experiments, which are much expensive than routine testing. Therefore, repeated testing can increase the detection rate of HBV-DNA-positive blood donors and reduce the expenses of blood donor re-entry. Moreover, using the model can better predict the status of HBV infection in non-repetitive reactive blood donors. Furthermore, this model may result in 15.49% (22/142) of negative blood donors being classified as positive. However, calculations revealed that among all non-repeat reactive blood donors from August 2010 to December 2022, only 283 blood donors may be misjudged, accounting for a small proportion of 87.94 per million (283/3218194) blood donors. The comparison of screening strategies indicated that using “two repeat discriminate tests + model predictions + re-entry tests” can achieve the best cost-effectiveness ratio. This method can also be adjusted based on the blood donor population in different regions, thus providing a basis for the re-entry strategy of HBV blood donors.

Through the risk factors analysis of HBV infection, high S/Co value, non-repeated HBV-DNA positive group, older age, and more donations were identified as risk factors for HBV infection. Although the TMA experiment was a qualitative test, a high S/Co value was positively correlated with discriminatory ability (cor = 0.27, *p* < 0.05). Older age is a risk factor for HBV infection, mainly because of its positive correlation with anti-HBc (cor = 0.07, *p* < 0.05), while other studies have indicated that the anti-HBc positivity rate increases steadily with age (19). However, repeat blood donors have a higher possibility of HBV infection when non-discriminated reactive results occur than non-repeat blood donors. The Mantel test results demonstrated a negative correlation between viral load and the number of blood donations; however, this was not statistically significant (*r* ≥ 0.05, *p* > 0.05), whereas a significant positive correlation was identified with grouping (*r* ≥ 0.1, *p* < 0.05). Furthermore, among the 435 blood donors in our study, 257 were repeat blood donors (≥ 3 times), accounting for 72.14% (189/262), 39.68% (25/63), and 39.09% (43/110) in each group, indicating that repeat blood donors are mostly non-repeated reactive blood donors. Similarly, in our previous studies, the interval between non-repeated reactive blood donors and the occurrence of NAT positivity was the longest, at 32.07 (±27.21) months, and there was a phenomenon of viral fluctuation (8). These findings suggest that blood donors with more donations are potentially non-reactive in repeat screening or discriminate tests and have lower viral loads, indicating a higher possibility of having a low viral load in occult HBV infection. This can also partially explain why repeated blood donors are at risk for HBV infection among non-discriminated reactive blood donors.

In summary, there was a specific proportion of HBV infection (65.52%, 285/435) among non-discriminatory reactive blood donors in the Blood Center of Zhejiang Province. Repeat testing methods can improve the detection rate of HBV-DNA, and constructing a predictive model for HBV infection status can significantly reduce the expense of re-entry and improve efficiency. Simultaneously, the infection status of repeat blood donors with non-repeat reactive results should be taken seriously and verified since they may be carriers of low viral load OBI.

## Data availability statement

The raw data supporting the conclusions of this article will be made available by the authors, without undue reservation.

## Ethics statement

The studies involving humans were approved by Ethics Committee of the Blood Center of Zhejiang Province. The studies were conducted in accordance with the local legislation and institutional requirements. The participants provided their written informed consent to participate in this study.

## Author contributions

DW: Data curation, Funding acquisition, Visualization, Writing – original draft, Investigation, Methodology, Conceptualization, Project administration, Software, Validation. YH: Investigation. MW: Investigation. YW: Investigation, Resources. JD: Investigation, Resources. JL: Investigation, Validation. WH: Writing – review & editing, Conceptualization, Resources, Supervision, Validation.
